# Nucleosome-coupled expression differences in closely-related species

**DOI:** 10.1186/1471-2164-12-466

**Published:** 2011-09-26

**Authors:** Yuanfang Guan, Victoria Yao, Kyle Tsui, Marinella Gebbia, Maitreya J Dunham, Corey Nislow, Olga G Troyanskaya

**Affiliations:** 1 Lewis-Sigler Institute for Integrative Genomics, Princeton University, Princeton, NJ, 08544, USA; 2 Department of Molecular Biology, Princeton University, Princeton, NJ, 08544, USA; 3 Donnelly Centre for Cellular and Biomolecular Research, University of Toronto, Toronto, Ontario M5S 3E1, Canada; 4 Department of Pharmaceutical Sciences, University of Toronto, Toronto, Ontario M5S 3M2, Canada; 5 Department of Genome Sciences, University of Washington, Seattle, WA 98195, USA; 6 Department of Molecular Genetics, University of Toronto, Toronto, Ontario M5S 1A8, Canada; 7 Banting and Best Department of Medical Research, University of Toronto, Toronto, Ontario M5G 1L6, Canada; 8 Department of Computer Science, Princeton University, Princeton, NJ, 08540, USA

## Abstract

**Background:**

Genome-wide nucleosome occupancy is negatively related to the average level of transcription factor motif binding based on studies in yeast and several other model organisms. The degree to which nucleosome-motif interactions relate to phenotypic changes across species is, however, unknown.

**Results:**

We address this challenge by generating nucleosome positioning and cell cycle expression data for *Saccharomyces bayanus *and show that differences in nucleosome occupancy reflect cell cycle expression divergence between two yeast species, *S. bayanus *and *S. cerevisiae*. Specifically, genes with nucleosome-depleted MBP1 motifs upstream of their coding sequence show periodic expression during the cell cycle, whereas genes with nucleosome-shielded motifs do not. In addition, conserved cell cycle regulatory motifs across these two species are more nucleosome-depleted compared to those that are not conserved, suggesting that the degree of conservation of regulatory sites varies, and is reflected by nucleosome occupancy patterns. Finally, many changes in cell cycle gene expression patterns across species can be correlated to changes in nucleosome occupancy on motifs (rather than to the presence or absence of motifs).

**Conclusions:**

Our observations suggest that alteration of nucleosome occupancy is a previously uncharacterized feature related to the divergence of cell cycle expression between species.

## Background

An organism's DNA contains numerous regulatory sequences that are used to modulate gene expression; yet DNA sequence alone does not explain why some regulatory sequences are functional while others are not. Because most genomic DNA (80% on average) is tightly packaged into nucleosomes [1], alternating nucleosome occupancy has been proposed as an important strategy to regulate gene expression since its initial discovery [2,3]. Indeed, higher expression levels are commonly associated with nucleosome depletion at promoters and other genomic locations, e.g. rDNA [1,4-6]. It has also been demonstrated that nucleosome occupancy affects the accessibility of DNA sequence motifs to transcriptional regulators; as a consequence different DNA sequences can display different nucleosome occupancy levels [1,4,7]. Further, motifs recognized and bound by active transcription factors are more likely to be nucleosome-depleted than those recognized by inactive ones [1,8-13]. Differential occupancy on many motifs has been observed in certain environmental conditions [14,15] and following environmental stresses [16]. However, it remains controversial whether changes of nucleosome occupancy [16] or their initial positioning [14] determines levels of gene expression.

Most previous studies have focused on measurements of average transcription levels and average nucleosome occupancy over regulatory regions. The one-to-one connection between the occupancy of individual motifs and the resulting effect on gene expression has been tested only for a small number of genes. A recent study demonstrated that nucleosome depletion at two cell cycle-regulated promoters, *CLN2pr *and *HOpr*, ensures periodic expression pattern of genes involved in cell-cycle progression [17]. These experiments clearly linked a specific expression pattern (cell-cycle periodicity) to nucleosome occupancy. The generality of this phenomenon for genes containing cell cycle regulating motifs remains to be tested through genome-wide experiments.

An average correlation between expression level and nucleosome occupancy at promoters across species has been reported [18], but it is not, however, clear how motif-specific nucleosome occupancy patterns affect the expression of individual genes across different species. To address this question, we sought an analysis approach that transcends the average expression level and targets the response at a specific class of motifs under specific conditions. Although predictions of nucleosome occupancy often assume that nucleosome positions are identical on conserved DNA sequences [19], experimental data is needed to test this assumption to better understand how nucleosome occupancy on motifs relates to phenotypic evolution. Such comparison across species can provide insight that augments ongoing efforts to define the relative contributions of *cis *and *trans *acting factors in phenotype divergence.

In this study, we determined the genome-wide nucleosome positions in the yeast *S. bayanus*, and compared these findings to patterns of gene expression during the cell cycle of *S. cerevisiae *and *S. bayanus*, two closely related *sensu stricto *yeast species. We show that changes in nucleosome occupancy on motifs are correlated with phenotypic divergence between species. In particular, our results show that nucleosomes provide a conspicuous genome-wide signature of MBP1 cell-cycle motif recognition in these two yeasts and this signature distinguishes which motifs result in periodic, cyclic expression patterns of the downstream genes. Although averaged expression level has previously been negatively linked to nucleosome occupancy at promoters [1,4-6], our data provide a high-resolution, genome-wide demonstration of how the interplay between nucleosome occupancy and motif content is related to a specific expression pattern (*i.e*. in the cell cycle) of individual genes. Conserved transcription factor binding sites are more likely to be nucleosome-depleted, suggesting that patterns of nucleosome occupancy may reflect conservation of regulatory circuits across species. Finally, our cross-species comparison of transcription factor binding sites and nucleosome occupancy patterns reveals that changes in nucleosome-motif interactions are correlated to expression divergence, i.e. despite their conserved presence, motifs that become nucleosome-occupied during evolution no longer regulate downstream gene expression.

## Results

### *Global nucleosome occupancy in *S. bayanus

We compared the closely related *Saccharomyces *species *S. bayanus *and *S. cerevisiae *to investigate how the interplay between nucleosomes and transcription factor binding motifs may affect downstream gene expression divergence. The two species are separated by 20 million years of evolution, a practical distance allowing us to investigate expression alterations between related genomes while still allowing for relatively unambiguous ortholog assignments. To map the genome-wide profile of nucleosome occupancy of *S. bayanus*, we used high-throughput, short read sequencing to detect nucleosome positioning as described in [1,15] (data deposited in the short read archive GSE24356). Applying a wavelet model [20], we identified 47,777 well-defined nucleosomes (Additional file 1, Test S1), consistent with published work [1], with 97.95% of the *S. bayanus *[21] genome assembly sequence covered (Additional file 2, Figure S1, Additional file 2, Figure S2).

As a quality control, we examined the *S. bayanus *genome for several features known to be present. Previous observations in *S. cerevisiae *and *S. bayanus *showed stereotypical nucleosome positioning relative to the start codon [1,18]. Consistent with previous observations of nucleosome depletion at start codons (ATG) in *S. cerevisiae *[8], in *S. bayanus *we observe nucleosome depletion centered at ~150 bp upstream of the start codon (Additional file 2, Figure S2B) and at ~90 bp downstream of the stop codon (Additional file 2, Figure S2C). In addition, we found nucleosome occupancy in *S. bayanus *peaks immediately downstream of the start codon, as observed in previous publications [18,22]. This confirms the technical quality of the sequencing data and provides an independent demonstration that the overall nucleosome occupancy pattern is conserved between *S. bayanus *and *S. cerevisiae *[18].

### Nucleosome depletion on cell cycle regulatory motifs predicts periodic expression for downstream genes in *S. bayanus *and *S. cerevisiae*

Previous studies have reported that, on average, nucleosomes are depleted over transcription factor binding motifs [1,8], leading to the suggestion that the active transcription factors are correlated to depleted nucleosomes on their target sites [1,8-12]. We wanted to test if this correlation holds true at the level of individual genes, i.e. if, at a given motif upstream of several genes that vary in their gene expression levels, the level of nucleosome occupancy is correlated to these differences in gene expression. To test this possibility, we analyzed cell cycle gene expression data from *S. bayanus *cultures synchronized with alpha-factor. Genes were ranked by their cell cycle expression periodicity as determined by Fourier transform (Additional file 2, Figure S3). The top motifs enriched in cell cycle-regulated genes were determined by a mutual information based algorithm, FIRE [23], with the consensus sequences [AGT]ACGCG[AT][ACG]A. This motif in *S. bayanus *maps to the *S. cerevisiae *cell cycle regulatory motifs for the transcription factor Swi4p (*e *= 2.01e-09) based on the motif comparative tool STAMP [24] and the AlignACE database [25]. The SBF (Swi4p-Swi6p) complex, in concert with MBF (Mbp1-Swi6p), is the principal transcriptional regulator of the yeast cell cycle [26].

**Figure 1:**
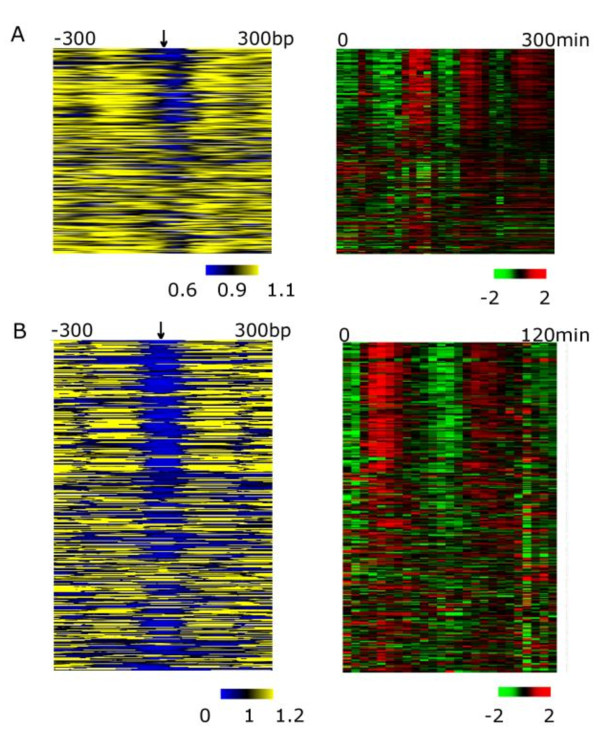
**Absence of cyclic expression pattern correlates with nucleosome occupancy in A. *S. bayanus *and B. *S. cerevisiae***. Genes with MBP1/SWI4 consensus sites within the upstream region of the coding sequence start site were ranked from top to bottom by accordance to their cell cycle periodicity. Nucleosome level from -300 to 300 bp of the motif positions (arrow) for those genes (left) were laid in parallel to their expression level (right).

Our analysis is centered on motifs so that we could directly observe the nucleosome occupancy on motifs without aligning start codons or aligning the +1 nucleosome [18]. We observed a striking pattern of nucleosome depletion on SWI4 motifs of periodically expressed genes (in this study upstream motifs are defined as motifs occurring in -600 to 0 bp), whereas, in contrast, nucleosomes tend to shield those motifs that occur in upstream regions of genes that do not show cell cycle regulation. This correlation is particularly apparent when nucleosome occupancy is examined alongside the expression data for the same set of genes: nucleosome depleted SWI4 motifs occur in genes that peak in expression at the G1/S stage of the cell cycle, reflecting the established function of Swi4p [26] (Figure 1A, p = 4.81E-05, using the Mann-Whitney *U *test, to accommodate the non-normal distribution of both factors). To verify that these patterns of nucleosome depletion were not simply a result of functional motifs occurring preferentially within the nucleosome depleted region of promoters, we repeated this analysis focusing only on the subset of genes (periodically expressed or not) with motifs in this 300 base pair nucleosome depleted region. Again, only cell cycle regulated genes show consistent nucleosome depletion over these regulatory motifs (Additional file 2, Figure S4). This indicates that the pattern of nucleosome occupancy reflects the active state of motifs on the individual gene level for this specific phenotype of cell cycle regulation. A secondary motif [AG]TAAACAA[AT] mapping to Fkh1p (*e *= 7.20e-08) was identified, and similar correlations between motif depletion and cell cycle expression were observed (Additional file 2, Figure S5). Because SBF-MBF is the primary driving factor in the cell cycle, our subsequent analysis focused on binding motifs of this complex.

To study the generality of this motif-nucleosome interaction on individual gene expression, we explored whether the motif-nucleosome interplay holds true in the related species, *S. cerevisiae*. Using cell cycle expression data from *S. cerevisiae *cells synchronized with alpha factor under similar conditions to our *S. bayanus *experiment [27], we identified genes displaying cell cycle periodicity (Additional file 2, Figure S3). The most significant motif we identified, with the consensus [AGT][AT]CGCGT[CT][AGT], corresponded to the MBP1 motif (*e *= 4.71e-07 using STAMP [28]). Mbp1p is a core member of the MBF complex with a consensus sequence very similar to that of SWI4 (*e *= 7.58e-05, STAMP), the motif we identified in *S. bayanus*. Consistent with our observations in *S. bayanus*, nucleosome depletion *(*data *from *[15]) at MBP1 motifs is correlated with periodic expression during the cell cycle (*p *= 6.27E-29, Mann-Whitney U test). Genes downstream of these depleted sites show peak expression at G1/S, the cell cycle stage regulated by SBF-MBF complexes [26] (Figure 1B). In contrast, genes with nucleosome-occupied motifs did not show a periodic pattern of expression. These results highlight that our observations can be generalized across species that have diverged by at least 20 million years.

**Figure 2:**
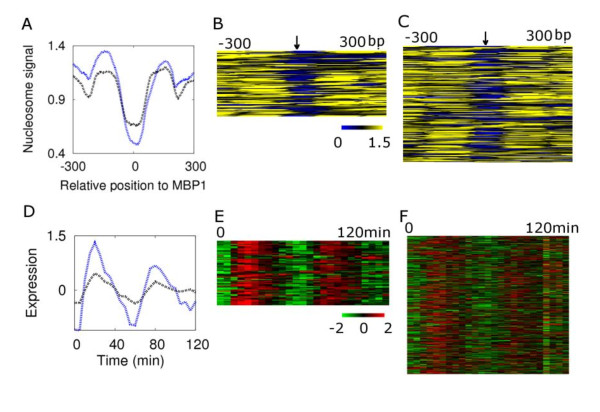
**Correspondence between MBP1 motif binding, nucleosome depletion and expression pattern**. **A**. Comparison of nucleosome occupancy between MBP1 bound motifs (blue line) [29] and unbound (black line) consensus sites CGCGT[CT] over the *S. cerevisiae *intergenic region. Comparison of nucleosome signals from -300 to 300 reveals that overall the bound (**B**) motifs (arrow) are more nucleosome-depleted than unbound (**C**) motifs although exceptions occur. **D**. Comparison of expression patterns during the cell cycle for genes with at least one bound MBP1 motif (blue line, as assessed by chromatin immuno-precipitation) in the region upstream of the start site, and genes (black line) that have an MBP1 consensus-match in the upstream intergenic region but that are not bound by the MBF protein complex. The cyclic expression patterns of genes with bound Mbp1p (**E**) are more apparent than the genes with an MBP1 motif present but without evidence of binding (**F**).

To directly examine whether the nucleosome-depleted motifs that are related to periodic expression in our study are correlated to transcription factor (TF) binding, we compared the nucleosome occupancy of Mbp1p bound and unbound motifs along with the expression patterns of their downstream genes [29]. The Mbp1p-bound sites are significantly more nucleosome-depleted than an average MBP1 consensus (CGCGT[CT]) site in the upstream region (Figure 2A-C) (*p <*0.00097, Welch's *t *test, for comparison of samples with unequal variance). We further investigated the gene expression patterns corresponding to the motifs occupied by Mbp1p or not. We categorized genes as follows; 1) Mbp1p-bound, containing an MBP1 consensus within the 600 bp upstream region of the gene, with the motif bound by the transcription factor Mbp1p or 2) Mbp1p-unbound, defined as having none of their upstream MBP1 sites bound by Mbp1p. Genes with the Mbp1p-bound sites (which are more nucleosome depleted) display more periodic expression (Figure 2D-F, *p *= 0.0082, Mann-Whitney's *U *test of the difference to cell cycle periodicity of the two groups). This directly supports that the cyclic expression of the downstream genes is related to Mbp1p binding and is reflected by nucleosome depletion on the motif sites.

### Motif preservation for orthologous genes is correlated with nucleosome depletion in *S. cerevisiae *and *S. bayanus*

**Figure 3:**
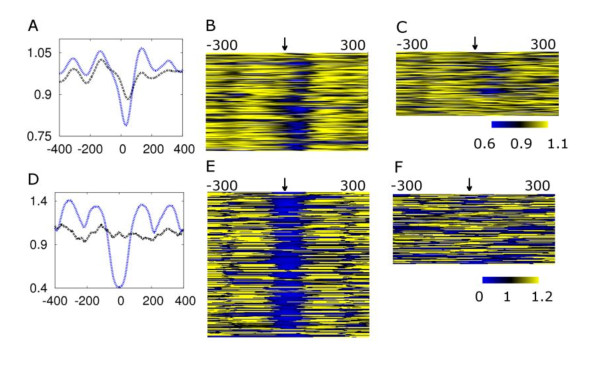
**Non-conserved cell cycle motifs are more nucleosome-occupied**. **A**. Genes containing the SWI4 DNA sequence binding consensus [AGT]ACGCG[AT][ACG]A in the upstream region in *S. bayanus *were divided into two categories, depending on motif occurrence in the orthologous upstream region in *S. cerevisiae*. The genes without the SWI4 consensus motif in *S. cerevisiae *(black line, **C**, arrow points to motif positions) tend to be more nucleosome-occupied on the motif position than the genes with conserved SWI4 motifs (blue line, **B**). **D**. The MBP1 DNA sequence consensus [AGT][AT]CGCGT[CT][AGT] observed in *S. bayanus *(similar to the MBP1 motif identified in *S. cerevisiae) *was divided into two categories: conserved (**E**) *versus *not present in *S. bayanus *(**F**). The genes with an MBP1 motif present in only *S. cerevisiae *(black line) are consistently more nucleosome occupied compared to the genes for which MBP1 binding sites are conserved in both species (blue line).

In both species, we observe that genes with cell cycle regulatory motifs are periodically expressed when the motifs are nucleosome-depleted. If nucleosome occupancy is an effective regulator of motif function, we would expect relaxed selection on nucleosome-occupied motifs compared to those that are nucleosome-depleted. This could manifest as differential loss rates among consensus sequences that are otherwise considered equal. We tested this possibility by examining the nucleosome occupancy pattern of motifs present only in one species compared to those that are conserved across both. We defined the 'absent' motifs as those that occur in the upstream sequence of one species but are absent within 200 bp around that position at the orthologous gene in the other species. The 'absent' group (*i.e*., motifs that only occur in one species) consistently showed significantly higher nucleosome occupancy than the conserved group (*i.e*., motifs that occur in both species). (Figure 3, *p *= < 0.0003 for conservation from *S. bayanus *to *S. cerevisiae, p *< 0.0001 for conservation from *S. cereviae *to *S. bayanus*, Welch's *t *test). While significant, this correlation represents only the overall tendency between nucleosome depletion and conservation of motifs, and exceptions are observed where some of the non-conserved motifs are also nucleosome-depleted. This result may partially explain the increased turnover rate of nucleosome-occupied DNA sequences observed in intergenic regions [19].

### Correlation between changes in gene expression and changes of nucleosome-motif interactions across species

**Figure 4:**
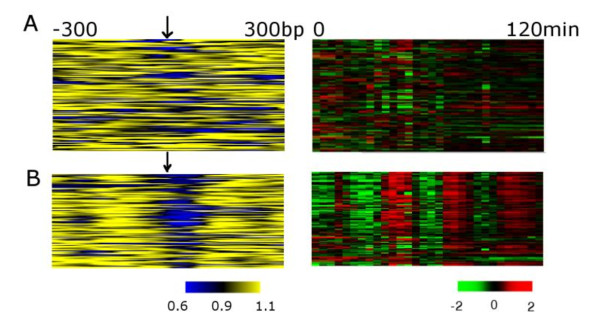
**Difference of cell cycle expression pattern across species achieved by either differences in motif content or occupancy by nucleosomes**. **A**. We identified MBP1 sites absent in *S. bayanus *but present in *S. cerevisiae*. The nucleosome signal around these sites (left) is compared against the expression profiles (right). **B**. The *S. bayanus *orthologous sequences of the functional motif sites from *S. cerevisiae *were ranked according to whether the downstream genes show a periodic expression pattern during the cell cycle. The nucleosome signal centered at the MBP1 motif (left) and the expression pattern of the downstream genes (right) was plotted.

The strong association between nucleosome depletion and the activity of a motif has an additional implication: changes in expression patterns across species should be correlated to either motif loss and gain or, alternatively through increased nucleosome occupancy of motifs in one species vs. nucleosome depletion in the other species. We therefore investigated the nucleosome occupancy levels at regulatory motifs in the upstream regions of orthologs that display changes in their cell cycle expression pattern between *S. bayanus *and *S. cerevisiae*. We defined the functional MBP1 motifs that control expression in *S. cerevisiae *as those that were upstream of 1) an open reading frame and 2) genes that were periodically expressed during the cell cycle. Because motif loss and gain are likely to be accompanied by changes in motif content and spacing [30], we restricted our comparison to motifs consistently present in the regulatory region of the orthologous genes in the two species, rather than considering only motifs with a conserved location within a particular promoter. We defined conserved motifs as those that appear at least once in the 600 bp upstream of the regulatory region of the orthologous gene. While this does not guarantee conservation of an exact motif (because its location can change across species), this approach ensures that the presence of any such nearby regulatory region is considered. Using these criteria, we divided the orthologous cell cycle expressed genes in *S. cerevisiae *into two categories: those that do not have an MBP1 motif in *S. bayanus *and those that have conserved this motif. We found that genes lacking MBP1 motifs (146 motifs) lack periodic expression in *S. bayanus *(Figure 4A), and the orthologous sequences corresponding to the *S. cerevisiae *motif positions are more nucleosome-occupied compared to those that are conserved (Figure 4, *p *= 0.0114, two tailed Welch's *t *test).

To investigate whether there is a correlation between nucleosome and motif interaction to cell-cycle expression conservation, we ranked the orthologous cell cycle regulated genes with conserved MBP1 motifs according to whether they are cyclically expressed in *S. bayanus*. Nucleosome depletion is a clear signal for those genes that remain periodically expressed in *S. bayanus *(Figure 4B, p = 7.2348E-05, Mann-Whitley *U *test). The conservation of periodic expression pattern is not an artifact of the existence of non-expressed genes: when we restrict the analysis to expressed genes (defined as the top 90% genes in expression level [31]), we observe the same pattern (Additional file 2, Figure S6). Similar results were obtained when testing the motif and gene expression conservation patterns in the opposite direction, *i.e*., from *S. bayanus *to *S. cerevisiae *(Additional file 2, Figure S7), supporting the generality of our observation and supporting the idea that motif gain/loss and changes in nucleosome-motif interactions contribute to alterations in gene expression patterns. This result suggests that alteration of nucleosome-motif interactions in the upstream regulatory region of genes is a biologically relevant phenomenon relating to gene expression divergence that is distinct from the loss of specific motifs in the regulatory region.

### Orthologous motif analysis on expression-nucleosome changes across species

**Figure 5:**
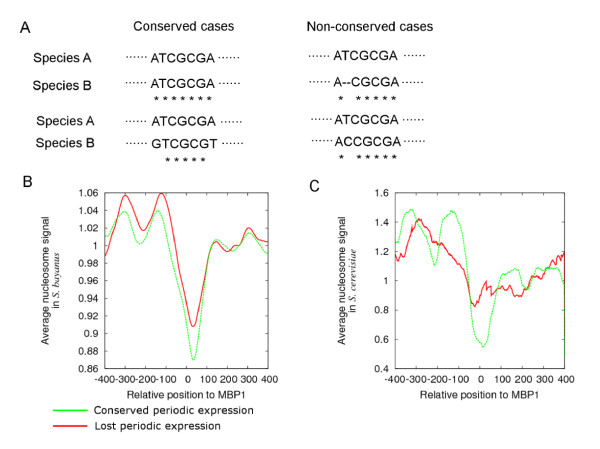
**Independently of motif movements, nucleosome occupancy is correlated to expression divergence**. **A**. Schematic of the alignment of the upstream regions to identify orthologous motifs between *S. cerevisiae *and *S. bayanus*. For the MBP1 binding site consensus [AGT][AT]CGCG[AT], a motif is considered to be conserved if the whole motif region is aligned and either the original motif is completely conserved, or it has changed into another form of the consensus. A motif is considered unconserved if the motif is not aligned or, despite complete alignment, the corresponding position does not constitute an MBP1 motif. **B**. All *S. cerevisiae *genes periodically expressed during the cell cycle and with an upstream MBP1 were first identified. Their orthologous motifs and genes in *S. bayanus *were then identified and only those genes with conserved motifs at aligned positions were considered. These *S. bayanus *orthologs were categorized into genes that show cyclic expression and those that do not. Nucleosome occupancy centered at these motifs of the two groups was plotted. **C**. We examined the conservation of expression patterns of *S. cerevisiae *from *S. bayanus *(a complementary comparison to **B**). The differential nucleosome occupancy levels of the two groups (conserved cyclic expression *vs*. non-cyclic expression) were depicted.

The analysis described above reveals combined effects of the presence of a motif and the levels of nucleosome occupancy on the conservation of cell cycle expression. We further tested the robustness of our analysis on aligned motifs. The upstream regulatory region of *S. cerevisiae *and *S. bayanus *were aligned and our subsequent analysis was restricted to the motifs at orthologous positions (Figure 5A). First, we considered those genes that are periodically expressed during the cell cycle in *S. cerevisiae *and which have an MBP1 motif in their upstream regulatory region. The orthologs of these genes with aligned MBP1 motifs were identified in *S. bayanus *and categorized into two groups: 1. those genes that preserved periodic expression during the cell cycle; and 2. those genes that no longer periodically expressed. We found that nucleosome occupancy is significantly lower in the first group than in the second group (Figure 5B, p = 0.045, Mann-Whitney *U *test).

We also performed the analysis in the reverse direction, considering the conservation of nucleosome occupancy from *S. bayanus *to *S. cerevisiae*. These orthologous genes in *S. cerevisiae *were categorized into those that exhibit a cyclic pattern during the cell cycle and those that do not. The motifs upstream of periodically expressed genes show greater nucleosome depletion than genes which are not regulated by the cell cycle (Figure 5C, p = 0.05, Mann-Whitney U test), suggesting that for some classes of genes nucleosome depletion alone can be correlated to expression changes independent of the movement of transcription factor binding motifs.

## Discussion

Our observations provide two important perspectives on the current understanding of the control of gene expression. First our study provides strong evidence that cell-cycle regulation of gene expression is correlated not only to transcription factor binding motifs, but also to nucleosome occupancy at these sites. This genome-wide observation is consistent with a recent observation that nucleosome-depletion could ensure cyclic expression in two cell cycle-regulated promoters, *CLN2pr *and *HOpr *[17]. It is important to note that the nucleosome data presented here are from an asynchronous culture, and therefore the reported occupancy reflects the integration of the nucleosome occupancy signal throughout the cell cycle. Nucleosome occupancy has been observed to fluctuate during the cell cycle in concert with periodic gene expression [32], and further studies at the level of individual genes and groups of genes will help to elucidate this cause-and-effect relationship. Indeed, deciphering specific cause-effect relationships between transcription factor binding and nucleosome depletion remains an important challenge.

Second, we show that changes in gene expression across species can be correlated to either loss of the regulatory motif *or *a change in nucleosome occupancy on conserved motifs. Compared to previous studies that focused on average expression level [18], we demonstrate that such correlation is significant for cell cycle expression regulation. Future work on nucleosome occupancy change on motifs between species and the resulting changes in gene expression may help, in part, to explain the disconnect between regulatory motif divergence and gene expression divergence. Specifically, it has been reported that most of the differences between species in transcription factor (TF)-binding motifs in yeasts and mammals have a very limited ability to predict gene expression divergence [33]. Our results demonstrate that alteration of gene expression patterns can be related to a combination of motif turnover and nucleosome occupancy. Therefore, studies of transcription factor binding motifs may benefit from considering nucleosome occupancy data in parallel.

Our observations on the evolution of cell cycle gene expression suggest that phenotype divergence is strongly related to changes of nucleosome-motif interaction. However, despite this general trend, not all expression pattern changes observed in our analysis are explained by changes in nucleosome occupancy or by alteration of motifs. This observation likely reflects the real underlying biological sophistication of gene expression control. As such, our results contribute a novel phenomenon that should be considered in studies aimed at understanding the divergence of gene expression during evolution and its impact on evolutionary change. The datasets provided with this study comprise a rich resource for additional analyses (e.g. those that incorporate additional genome-wide, high-resolution data) to address these and related questions.

## Conclusions

Through analyzing nucleosome occupancy pattern and cell cycle expression divergence between two yeast species *S. bayanus *and *S. cerevisiae*, we found that many changes in cell cycle gene expression patterns across species can be correlated to changes in nucleosome occupancy on motifs (rather than to the presence or absence of motifs). This observation suggests that alteration of nucleosome occupancy is a previously uncharacterized feature related to the divergence of cell cycle expression between species.

## Methods

### 1. Nucleosome data preparation

#### Nucleosome sequencing data preparation

*S, bayanus*, also referred to as *S. bayanus var uvarum*, (623-6C) was obtained from Mark Johnston at the University of Colorado, Denver. In brief, yeast cells were grown in YPD at 27°C and harvested at mid-log phase (OD = 0.6). Nucleosomal DNA was isolated using protocols modified from [1] where mono-nucleosome sized DNA fragments were gel-purified and sequenced on the Illumina platform to produce 36 nt reads.

#### Mapping nucleosome reads

*S. bayanus *reference genome [21] was obtained from SGD (http://www.yeastgenome.org). We aligned nucleosome short reads (in total 11,841,061 reads) to this reference genome using Maq 0.7.1 (Mapping and Assembly with Quality) [34]. This resulted in the number of matched reads at each chromosome location for sense and antisense strands. Nucleosome positions were defined according to [6]. This nucleosome signal was further normalized by the ratio of the log value to the log of the median of the genome. The genome median is 1.

#### Modeling nucleosome positions for visualization

To visualize approximate nucleosome positions in *S. bayanus*, we applied NPS (Nucleosome Positioning from Sequencing) [20] to predict the positions of nucleosomes based on a wavelet model. Using FindPeaks 4.0, we converted the nucleosome signals based on the triangle distribution Additional file 2, Figure S2).

#### Coverage calculation

Given the estimated nucleosome positions, we calculated the percentage of nucleosome coverage of each contig by finding the ratio of nucleosomes within each contig (in bps) to the total length of each contig (in bps). In general, contigs with a reasonable size are well-covered, while very short contigs are likely to be poorly covered and also do not contain open reading frames. We therefore ranked the contigs by their coverage Additional file 2, Figure S1), and found 20% coverage (as determined by the nucleosome center regions by NPS [20]) to be a reasonable cutoff for well-covered contigs. To quantify the well-covered regions, we calculated the percentage of all contigs with greater than 20% nucleosome coverage (in bps) against the size of the entire genome. The open reading frames in these well-covered contigs were included in the following analyses. Two genes with unusually high numbers of mapped reads (> 100 times the genome average) were excluded from the analysis, and this resulted in the final 4935 genes included in the study.

### 2. Cell cycle data analysis

#### Cell cycle data and normalization

We acquired *S. cerevisiae *cell-cycle data from the alpha factor synchronization study described in Pramila *et al*. 2006 [27] and *S. bayanus *alpha factor synchronization cell-cycle data described in [35] (available at GSE16544). Genes with more than half of the values missing were removed from the analyses and other missing values were imputed with KNNimpute [36], with *K *= 10, Euclidean distance. Genes with duplicate measurements were averaged. For each gene in the cell cycle data, the expression values were centered so that the average over the time course equals to 0. The orthologous gene matches of *S. bayanus *and *S. cerevisiae *were obtained from [37].

#### Identification of cell cycle frequency and the motifs that drive the cell cycle

For *S. bayanus*, we directly applied Fourier transform [38] to identify the major cell cycle frequency (Additional file 2, Figure S3A). Fourier transform decomposes the time-course expression into different frequencies. For *S. bayanus*, the frequency with maximum amplitude over all genes is a noise signal because the canonical cell cycle regulated genes such as MBP1 do not show maximum amplitude at this frequency. The frequency with the secondary maximum amplitude is the cell cycle frequency. Genes were ranked according to their differences from the cell cycle frequency.

For *S. cerevisiae*, a set of cell-cycle regulated genes were identified in [27]. We identified the major cell cycle frequency based on the expression pattern of this set of genes (Additional file 2, Figure S3B) using Fourier transform. All genes were ranked according to their difference from the cell cycle frequency.

For both datasets, this ranking was used to identify the motifs most enriched in the cell cycle-regulated sets using FIRE [23]. Motifs identified by FIRE [23] were mapped to the AlignACE database by STAMP [28]. Often each motif could be mapped to a set of related motifs, for example, STB1, SWI4 and MBP1, in which case we labeled the motif with the best match. The positions of these motifs were then scanned throughout the whole genome using FIRE [23].

For each gene, we applied Fourier transform and identified the frequency with the maximum amplitude. The difference *d *of this frequency and the cell cycle frequency is calculated. The distribution of *d_g _*is plotted for all genes *g*, and the inflection point of this distribution is identified with the difference value of *d_inflection_*. Cell cycle-regulated genes were defined as those whose *d_g _*is smaller than *d_inflection_*, which indicates their pattern of expression is similar to cell cycle frequency. Conservation of cell cycle-regulation is defined as in both species, the periodicity difference is smaller than that of the inflection point.

The nucleosome signal around motifs and the cell cycle expression data were visualized in MeV4.6 by matching genes.

### 3. ChIP-chip data

We obtained Mbp1p binding sites data from [29], and mapped these sites to the *S. cerevisiae *May 2006 genome assembly, so as to match the nucleosome data we used. The genome-wide MBP1 positions were scanned using FIRE [23] based on the consensus defined in the AlignACE database.

### 4. Statistical analysis of changes in nucleosome occupancy

#### Calculating statistical significance between nucleosome occupancy and cell cycle periodicity

For each gene, we define its closeness to cell cycle periodicity based on the difference of its major frequency from the cell cycle frequency. For all the matches between a motif and its downstream gene, we used the Mann-Whitney U test to calculate the significance of the similarity of the distribution between the nucleosome signal and the gene's closeness to cell cycle periodicity.

#### Calculating statistical significance of nucleosome occupancy pattern on motifs

We used the following method to calculate the statistical significance when comparing nucleosome occupancy between conserved and non-conserved motifs, or between motifs driving periodic expression and non-functional motifs. For all occurrences of a motif, we identified the trough (lowest) position of the average nucleosome signal on these occurrences. The individual values on this trough position were recorded. The *p *value was calculated using Welch's *t *test.

For analysis of motifs that are conserved during evolution between *S. cerevisiae *and *S. bayanus*, we used alignment from [39]. Only motifs that occur within the orthologous regions are considered in such analysis.

## Authors' contributions

YG and VY carried out the computational analysis. KT and MG carried out the nucleosome experiments. CN, MJD and OGT designed the experiments. YG, CN, MJD, OGT wrote the manuscript. All authors read and approved the final manuscript.

## Supplementary Material

Additional file 1 **nucleosome positions**. Nucleosome positions identified by NPS. Click here for file

Additional fire 2 **Supplementary figures**. supplementary figures and their descriptions. Click here for file
