# Supplementary material for: Nucleosome-coupled expression differences in closely-related species
Source: BMC Genomics. 2011 Sep 26;12:466. doi: 10.1186/1471-2164-12-466 (PMC3209474; doi:10.1186/1471-2164-12-466)
Supplement: Additional fire 2 — Supplementary figures. supplementary figures and their descriptions. [file 1471-2164-12-466-S2.DOC]

**Figure S1. Nucleosome coverage on *S. bayanus* contigs.** Contigs were ranked by their % coverage of nucleosomes identified through NPS ^1^. Using 0.2 as cutoff for regions identified to be nucleosome centers by NPS, 97.95% of the entire assembly length was included in the analysis.

**
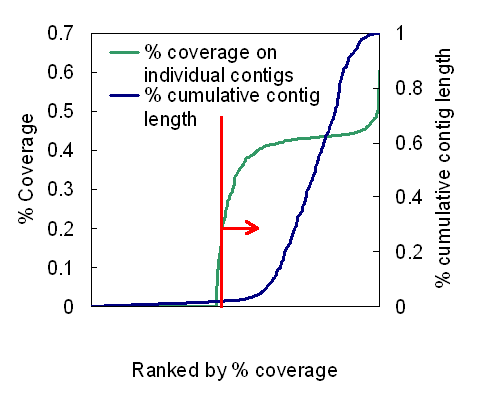
**

**Figure S2: Genome-wide pattern of nucleosome occupancy in *S. bayanus*. A.** Distribution of nucleosomes in a representative region at *S. bayanus* contig 563, 10,070-17,950 bps. Periodic nucleosome signals as matched tags on the chromosome location were identified by a wavelet model after matching sequencing tags to the *S. bayanus* reference genome. Nucleosome depletion is often observed immediately upstream of open reading frames, as observed in *S. cerevisiae*. **B.** Normalized nucleosome signal over all ORFs from -400 bp to +400 bp with respect to the ATG. A deep trough appears 145bp upstream of the ATG, with a sharp peak of nucleosome occupancy at 13 bp. **C.** Average nucleosome signal of all ORFs from -400
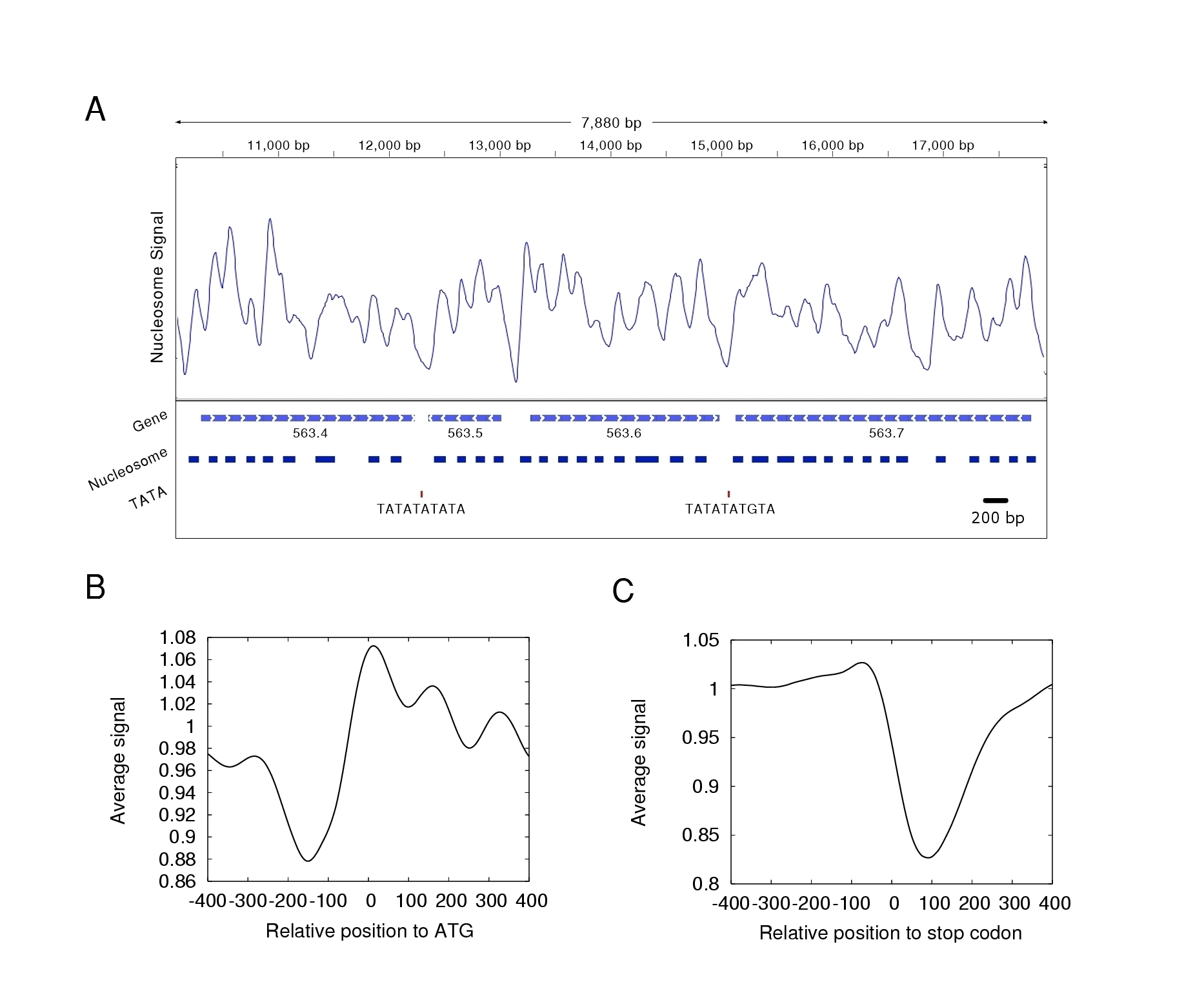
bp to +400 bp with respect to the stop codon. A peak appears at 73 bp upstream of stop site and a trough at 91 bp downstream.

**Figure S3. Cell cycle frequency identification by Fourier transformation.** We applied Fourier transform to the expression pattern of every gene. The summed amplitudes of frequencies are plotted. For *S. bayanus* (**A**), the frequency with secondary maximum amplitude is the cell cycle frequency, because the majority of the canonical cell cycle-regulated genes fall into this frequency. For *S. cerevisiae* (**B**), we plotted the frequency distribution of a set of cell cycle-regulating genes in *S. cerevisiae*. The frequency with maximum amplitude is the cell cycle frequency.

**
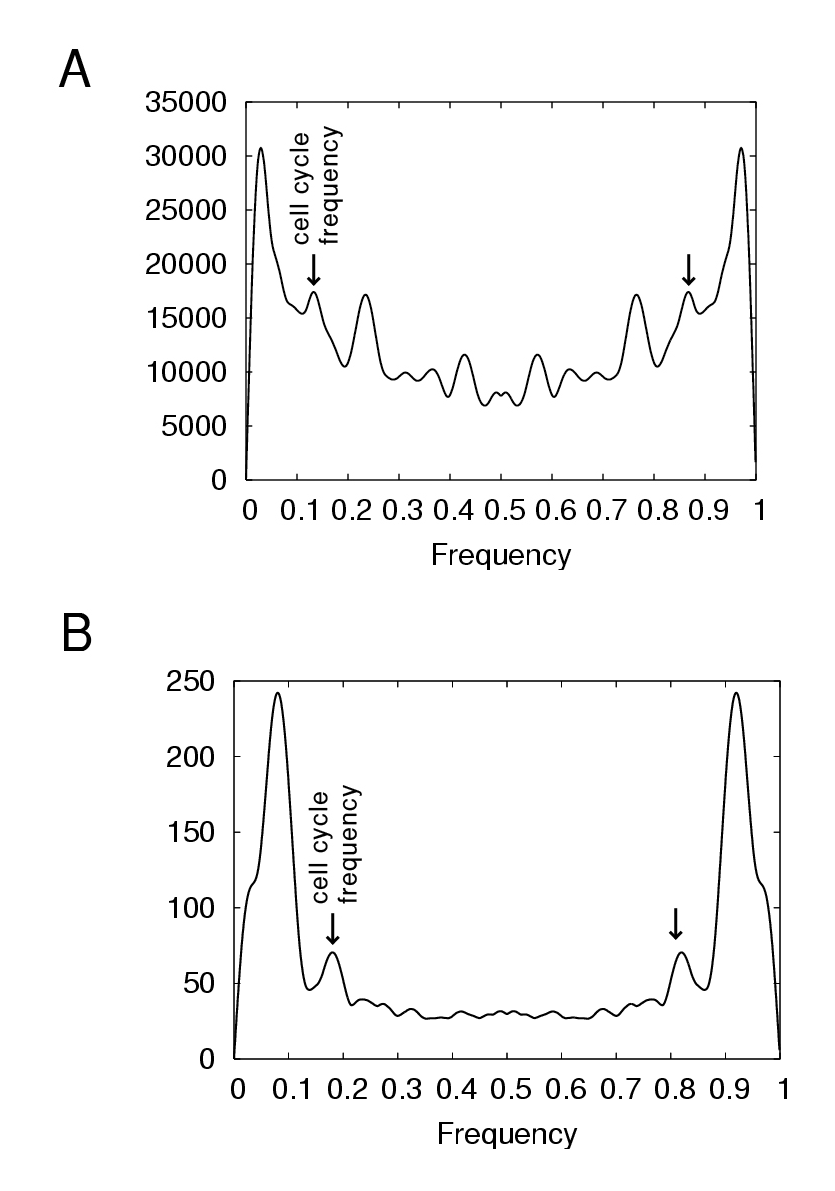
**

**Figure S4 Nucleosome-motif interaction in *S. bayanus* is not a mere effect of promoter occupancy.** In this analysis, we restricted the motif position to -300 ~0 bp upstream of start codon. Nucleosome (left) depletion marks the functional SWI4 motifs in regulating the transitions of gene expression (right) (*p* = 1.28E-12, Mann-Whitney *U* test). Genes with SWI4 consensus sites within 300 bp upstream were ranked by accordance to cell cycle periodicity. The upper portion was marked by depletion of nucleosome and cycling expression, whereas the lower portion shows random patterns.

**
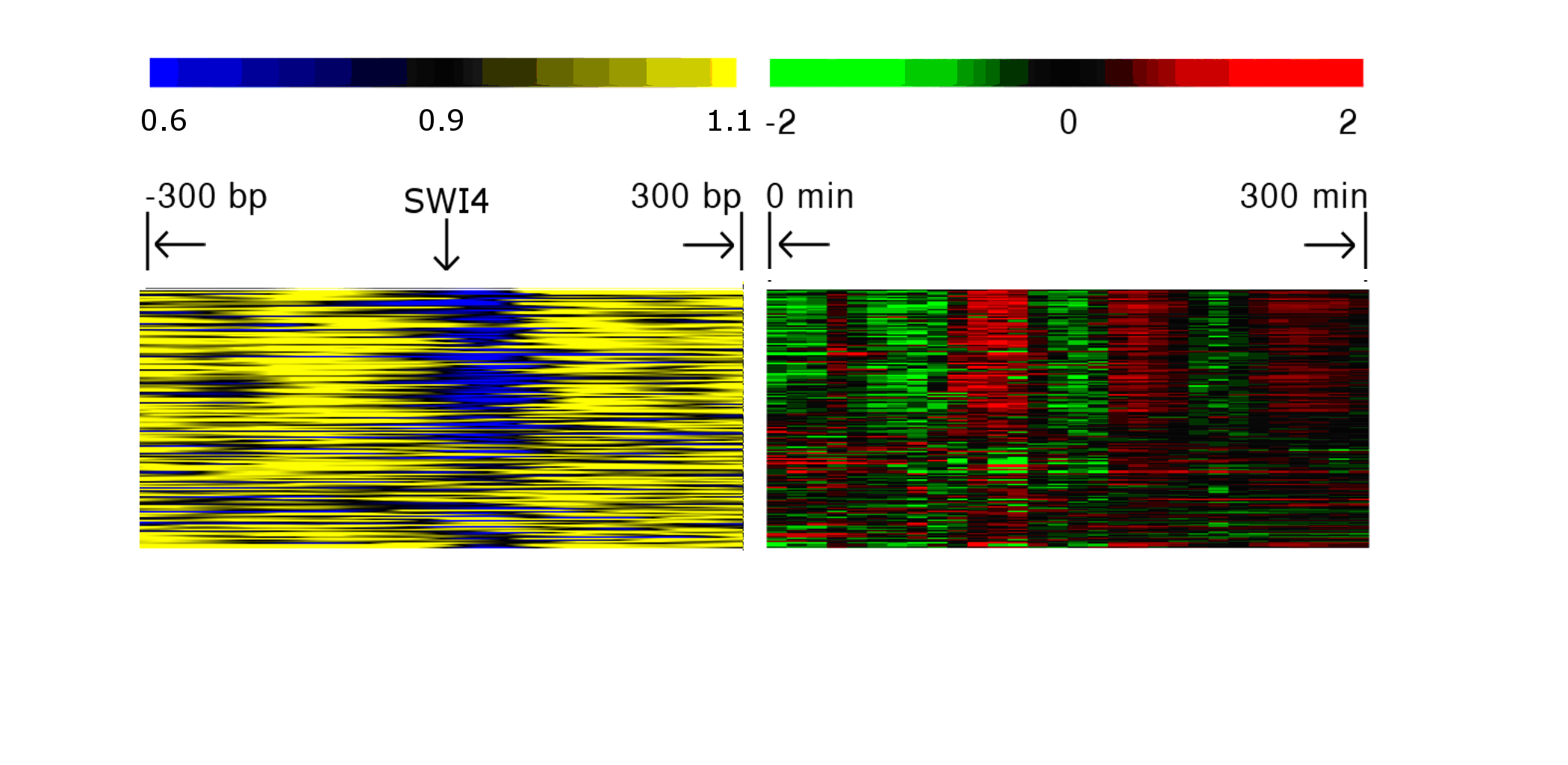
**

**Figure S5. Nucleosome (left) depletion weakly indicates the functional FKH1 in regulating the transition of gene expression at G2/M phase (right).** A secondary motif FKH1 was identified in *S. bayanus* cell cycle-expressed genes. Nucleosome depletion is correlated to cell cycle expression (*p* = 5.4E-06, Mann-Whitney *U* test).

**
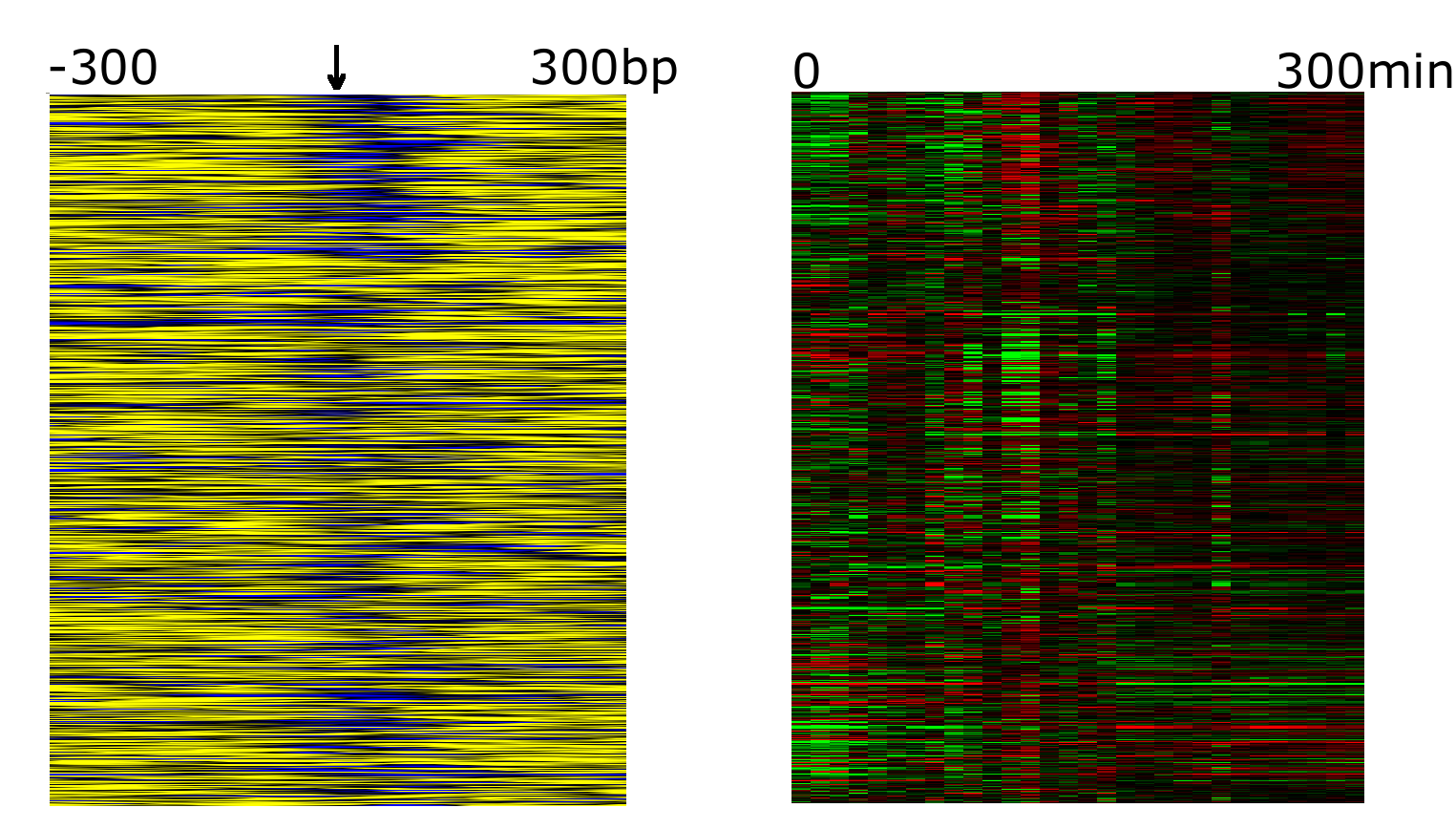
**

**Figure S6. The correlation between nucleosome occupancy on motifs and the cell cycle expression pattern of downstream genes is not an artifact of gene expression level.**  The analysis setup resembles Figure 1 but only includes expressed genes, defined as the top 90% expressed genes. Nucleosome (left) depletion marks the functional SWI4 motifs in regulating gene expression (right) during cell cycle. All Swi4p binding sites were identified by scanning the entire *S. bayanus* genome for the consensus SWI4 sequence. Genes with SWI4 consensus sites within 600 bp upstream of the coding sequence start site were ranked by their accordance to cell cycle periodicity. The most periodic genes show nucleosome depletion at the SWI4 consensus site, while the SWI4 motif tends to be nucleosome occupied in the less periodic genes.


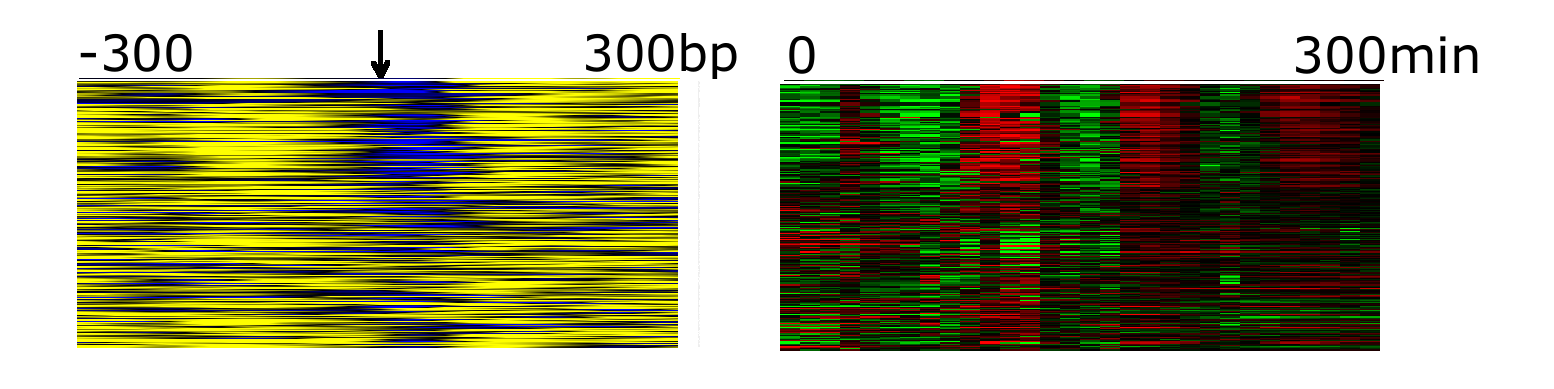


**Figure S7. Loss of cell cycle expression pattern across species (from *S. bayanus* to *S. cerevisiae*) achieved by either absence of motif or motif occupancy by nucleosomes.** The *S. cerevisiae* orthologous sequences of the functional motif sites from *S. bayanus* were divided according to whether the motif is absent or present in *S. cerevisiae*. **A.** When the functional motif is present only in *S. bayanus*, genes do not show cycling patterns of expression during the cell cycle and the orthologous sites show higher nucleosome occupancy compared to the conserved motifs. **B**. For the conserved motifs, genes selectively show a cyclic expression pattern depending on whether or not the corresponding motif is nucleosome-occupied.

***
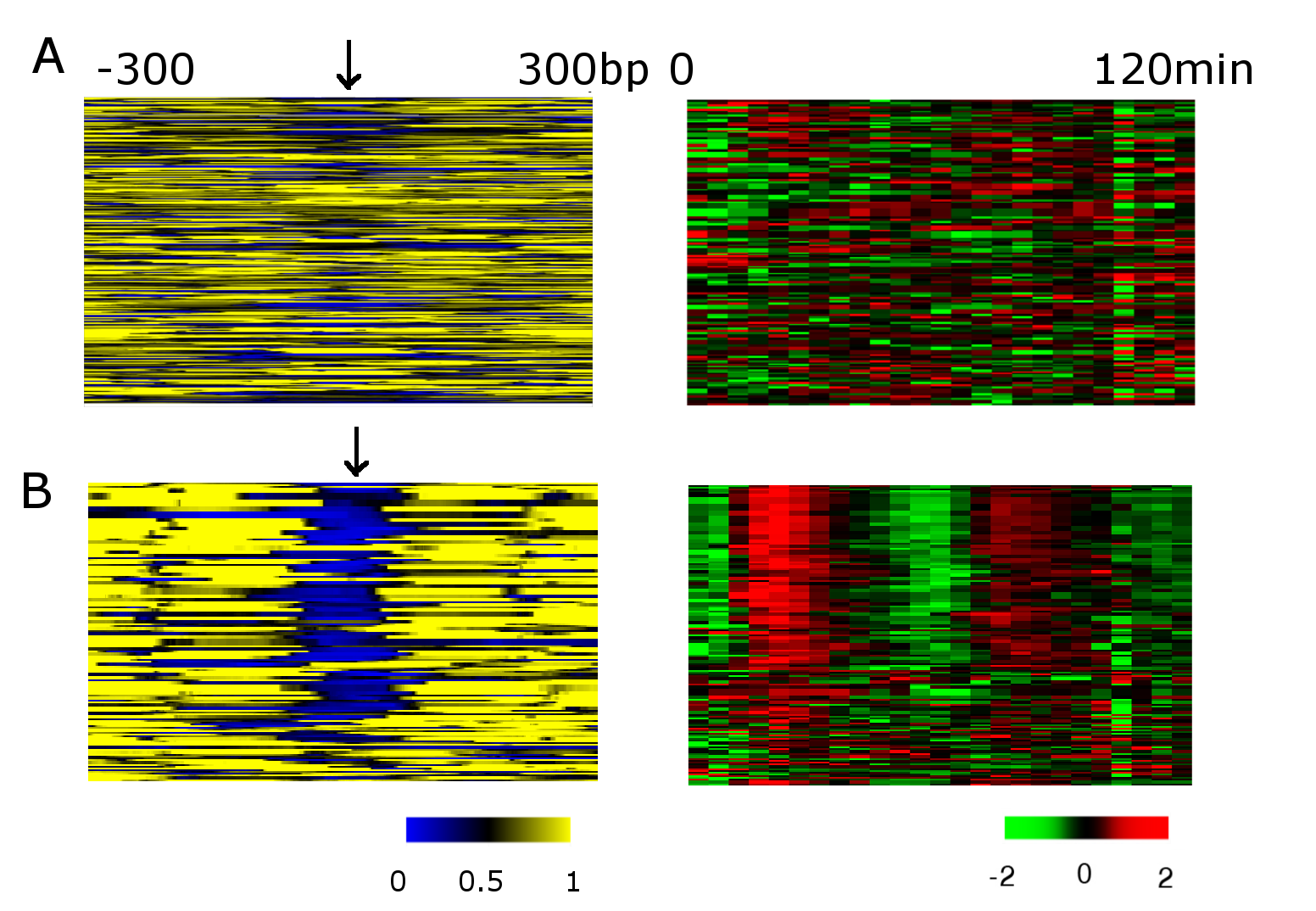
***
